# Toxicity of Nanomaterials

**DOI:** 10.1155/2015/521014

**Published:** 2015-05-20

**Authors:** Haseeb A. Khan, Rishi Shanker

**Affiliations:** ^1^Department of Biochemistry, College of Science, King Saud University, Riyadh 11451, Saudi Arabia; ^2^Institute of Life Sciences, School of Science and Technology, Ahmedabad University, Ahmedabad 380009, India

This decade has seen revolutionary developments in the field of nanotechnology with newer and diverse applications of nanoparticles (NPs) appearing every day. However, there are limited data about the toxicity of nanoparticles and their fate in biological systems. Inhalation, ingestion, and dermal penetration are the potential exposure routes for nanoparticles, whereas particle size, shape, surface area, and surface chemistry collectively define the toxicity of nanoparticles. Increased production and intentional (sunscreens, drug delivery) or unintentional (environmental, occupational) exposure to nanoparticles are likely to increase the possibilities of their adverse health effects. It is crucially important that novel nanomaterials must be biologically characterized for their health hazards to ensure risk-free and sustainable implementation of nanotechnology.

Although NPs have always occurred in nature, the latest developments are in the production and use of engineered NPs which are finding applications in a wide range of areas including cosmetics, medicine, food and food packaging, bioremediation, paints, coatings, electronics and fuel catalysts, and water treatment. The drugs encapsulated into nanoparticles result in a clump-free, stable, and water-soluble material due to a very large surface to volume ratio. Nanoparticle-mediated drug delivery systems are being developed for preventive treatment of the oxidative damage implicated in various neurodegenerative diseases such as Alzheimer's disease, Parkinson's disease, and Wilson's disease. Novel nanomaterials are also being explored for potential therapeutic and diagnostic applications in cancer treatment and diagnosis where the nanoscale properties facilitate the entry and intracellular transport to specific target sites. Nanomaterials also hold great promise for reducing the production of wastes and industrial contamination and improving the efficiency of energy production and use. The potential for nanotechnology is believed to be practically limitless and the potential for profiting from creating and marketing these advances is the driving force behind an incredibly rapid rush to deliver these applications to the marketplace.

However, the production, use, and disposal of manufactured NPs lead to discharges into air, soils, and aquatic systems. Therefore, it is crucial to investigate their transport into and through the environment and their impacts on environmental health. The indiscriminate use of engineered NPs with unknown toxicological properties might pose a variety of hazards for environment, wildlife, and human health. Our knowledge of the harmful effects of nanoparticles is still very limited and at present no specific regulations have been developed for NPs usage. Since the nanomedicine and nanotoxicology are two sides of the same coin, the worth of this coin depends on its prudent use. There is potential risk for the exposure of humans and the environment to nanoparticles throughout their life cycle, starting from manufacture to disposal. Accidental spillages or permitted release of industrial effluents in waterways and aquatic systems may result in direct exposure to nanoparticles of humans* via *skin contact, inhalation of water aerosols, and direct ingestion of contaminated drinking water or particles adsorbed on vegetables or other foodstuffs. The small size of NPs facilitates their uptake into cells and translocation across epithelial and endothelial cells. NPs may travel to other places in body and interact with tissues prolonging their stay in the body. There is predominant accumulation of NPs in organs with high phagocytic activity, mainly in liver, kidney, and spleen. Toxic effects have been documented at the pulmonary, cardiac, reproductive, renal, cutaneous, and cellular levels. A precautionary approach is required for individual evaluation of new nanomaterials for potential risks to health and environment associated with the application of these nanomaterials. Although current toxicity testing protocols may be applicable to identify harmful effects associated with nanomaterials, research into new methods is necessary to address the special properties of nanomaterials.

This special issue is a collection of 16 peer-reviewed original research and review articles, describing the toxicological properties and biocompatibility of the commonly used nanomaterials. Most of the articles in this special issue are devoted to carbon nanomaterials. Carbon is nonmetallic with peculiar property of being able to bond with itself and a wide variety of other elements. Natural carbon exists in two different forms: graphite and diamond. Three additional forms of carbon including fullerenes, nanotubes, and graphene were discovered between 1985 and 2004. Owing to their nanoscale structure, these forms of carbon were classified as carbon nanomaterials. Graphene is a two-dimensional, atomic-scale, and hexagonal lattice in the form of a flat carbon sheet. It is the basic structural element of other allotropes, including carbon nanotubes and fullerenes. Fullerenes are spherical carbon-cage molecules with sixty or more carbon atoms and measure about 0.7–1.5 nm in diameter. Carbon nanotubes (CNTs) are seamless cylinders of one or more layers of graphene with open or closed ends. Carbon nanotubes are the third most widely used materials due to their significant properties like thermal stability, lower mass density, and high mechanical strength. CNTs have now been incorporated in more than 35 products for the various applications such as sporting goods, automotive vehicles, clothing, electronics, and computers. The widespread use has enhanced their annual production globally from 300 tons in 2006 to about 4500 tons in 2011. The lipophilic nature, higher reactivity, and long life span of CNTs can adversely impact human health and environment. CNTs can easily pass the biological barriers and get distributed into the cellular and subcellular organs, but their toxicity is concentration dependent. It has been observed in the mice that inhalation exposure can cause pleural fibrosis in some cases; it is also reported that it shows the toxicological activities like asbestos and leads to the mesothelioma and granuloma. The toxicity studies in vitro in cell lines demonstrated reactive oxygen species (ROS) generation, inflammatory responses, DNA-damage, and oxidative damage of proteins. The ecotoxicity of CNTs has been assessed in bacteria, protozoans, crustaceans, and fishes. For example, CNTs affect the ingestion and digestion in ciliated protozoa, ultimately reducing bioavailability of free iron in the marine environment. There is a need to develop the methodologies and techniques to detect, characterize, and determine the transformation of CNTs in environment and biological systems. The rise in the use of CNTs demands health and safety standards for CNT manufacturing operations that potentially generate airborne particulate matter.

Some articles in this issue describe the toxicities of metal oxide NPs including three articles devoted to the toxicity of iron oxide NPs and one on the toxicities of nickel and cerium oxide NPs. One article reported the toxicity and tolerance of zerumbone-loaded nanostructured lipid carrier in a mouse model. There are two review articles in this special issue that focus on the general aspects of advantages and disadvantages (toxicity) related to applications of different types of NPs. Although there is a general recognition that nanotechnology has the potential to advance science and quality of life and to generate substantial financial gains, a number of reports have also suggested that potential toxicity should be considered as a primary concern for safe use of nanoparticles. The current knowledge of the toxic effects of nanoparticles is relatively limited. A precautionary approach is therefore required for evaluation of potential risks to health and environment associated with the use of nanomaterials. Although current toxicity testing protocols may be applied to identify harmful effects of NPs, research into new methods is required to address the special properties of nanomaterials. It is crucially important to assess their safety for sustainable implementation of nanotechnology with its full potential.

*Haseeb A. Khan* *Rishi Shanker*

